# The impact of temperature changes on the health vulnerability of migrant workers: an empirical study based on the China family panel studies

**DOI:** 10.3389/fpubh.2025.1519982

**Published:** 2025-02-26

**Authors:** Ting Liang, Zilin Ai, Hui Zhong, Mengyan Xiao, Mengzhou Xie, Xiaoli Liang, Liang Li

**Affiliations:** ^1^School of Chinese Medicine, Hunan Brain Hospital, Hunan University of Chinese Medicine, Changsha, Hunan, China; ^2^Hunan Engineering Technology Research Center for Medicinal and Functional Food, Changsha, Hunan, China; ^3^Key Laboratory of TCM Heart and Lung Syndrome Differentiation & Medicated Diet and Dietotherapy, Changsha, Hunan, China; ^4^Department of Political Science and Public Administration, Guangxi Normal University, Guilin, Guangxi, China; ^5^School of Dental Medicine, Hunan University of Chinese Medicine, Changsha, Hunan, China

**Keywords:** temperature change, migrant workers, health vulnerability, psychological burden, socio-ecological model

## Abstract

**Introduction:**

Migrant workers constitute a significant portion of China’s workforce, and their health directly affects labor supply and economic stability. Health vulnerability plays a crucial role in shaping the well-being of migrant workers, yet its determinants, particularly the impact of temperature change, remain underexplored. This study, based on the socio-ecological model, investigates how temperature variations influence the health vulnerability of migrant workers in China.

**Methods:**

Using data from 2020, this study quantifies health vulnerability and examines the impact of temperature fluctuations across different seasons. Robustness checks, including dependent variable substitutions and model modifications, ensure the reliability of the findings. Furthermore, a mechanism analysis is conducted to explore the underlying pathways through which temperature change affects health vulnerability.

**Results:**

The findings reveal that rising temperatures in spring, summer, and winter significantly exacerbate the health vulnerability of migrant workers, while increasing autumn temperatures mitigate it. Mechanism analysis identifies heightened psychological burden as a key channel through which temperature change worsens health vulnerability. Additionally, generational differences emerge: older migrant workers are more adversely affected by elevated spring temperatures, whereas younger workers exhibit greater sensitivity to rising summer temperatures.

**Discussion:**

These results underscore the necessity of targeted health interventions and adaptive labor protection policies. By highlighting the seasonal and generational disparities in the effects of temperature change, this study offers theoretical and empirical support for enhancing the resilience of migrant workers to climate variations. The findings provide valuable insights for policymakers in designing strategies to safeguard the health and stability of the migrant workforce.

## 1 Introduction

The combustion of fossil fuels and the accumulation of greenhouse gases in the atmosphere have resulted in a continuous rise in global temperatures (1). The escalating trend of global climate warming is an irrefutable reality, with rising temperatures profoundly affecting social and health systems globally, especially as the incidence of extreme weather events has markedly increased (2). Temperature variations, as a key manifestation of climate change, are progressively affecting human health and well-being in multifaceted and profound manners. Temperature variations are strongly linked to the morbidity of numerous diseases and influence physiological processes (3, 4), including DNA methylation and metabolic function, consequently intensifying the emergence of various illnesses (5, 6). Despite the human body’s fundamental thermoregulatory capabilities (7), extended exposure to elevated temperatures may compromise cerebral function (8) and may result in irreparable neurological damage (9). Nonetheless, temperature fluctuations do not impact all groups uniformly (10). The combined influence of climatic and socioeconomic has intensified the health vulnerability of some populations. During China’s urbanization and industrialization, numerous rural laborers have liberated themselves from land constraints, traveling to cities in pursuit of non-agricultural employment opportunities. Owing to the limitations of the urban–rural dual structure, the majority of migratory workers find it difficult to transition to urban citizenship. This group, encumbered by rural household registration and urban employment status, frequently suffers from uncomfortable working and residential conditions and insufficient safeguarding of their rights, all in the quest for societal advancement (11, 12). As for 2023, the population of migrant workers in China has neared 300 million, representing over one-third of the country’s labor force (13). Despite their substantial contributions to non-agricultural industries, overall social output, and economic progress in China (14), migrant workers frequently experience elevated job pressure, inadequate rest, poor nutrition, and precarious employment, whose working environments and living situations require continuous improvement (11, 15). Migrant workers encounter the problem of reconstructing their human and social capital in metropolitan environments due to the disruption of their original familial resources and social connections post-migration, making it difficult for them to establish stable roots in cities in the short term (16). Migrant workers, restricted by the urban–rural dual system, can primarily receive benefits from medical insurance schemes like the New Rural Cooperative Medical System exclusively in their registered hometowns. In their workplaces, they have several obstacles in obtaining medical services, social security, and health management resources comparable to those accessible to metropolitan inhabitants. Temperature variations intensify the health vulnerabilities of this demographic, highlighting structural issues and institutional blind spots in the allocation of social resources. Vulnerability is not only a reflection of health issues but also indicative of the efficacy of social resource allocation (17). The health susceptibility of migrant workers reflects the efficacy of resource allocation within the social framework. It signifies both the inefficiency of resource allocation for this group and their vulnerability and incapacity to manage environmental and social stresses (18). In China, the investigation of health concerns among migrant workers is shaped by the “healthy migrant effect” and the “salmon bias,” whereby migrants in poor health often return to rural regions, while those who remain in urban areas typically demonstrate superior health, resulting in a “health paradox” within the transient population (19). This bias results in inadequate emotional and material support for migrant workers at the macro level by the government, while simultaneously underscoring the substantial health decline among this demographic (20). These factors further intensify the health vulnerability of migrant workers. Migrant workers encounter many obstacles in obtaining equivalent healthcare benefits as urban residents (21–23), sometimes results in untreated health issues, exacerbating psychological stress. Anxiety and depression are common among migrant workers, arising from both life pressures and inadequate support systems (24, 25). Migrant workers, as a vulnerable demographic confronting social and environmental adversities, are a population of considerable scholarly significance and contemporary importance. Their health vulnerabilities necessitate additional investigation. This study will examine how temperature changes intensify the health vulnerabilities of migrant workers through intermediary mechanisms including psychological stress, revealing the complex health challenges stemming from the convergence of social context and climate change. This research possesses substantial theoretical significance—introducing a novel perspective on climate change and health vulnerability—while also bearing considerable practical implications—furnishing empirical evidence for the development of more focused health protection and social policies, thus offering scientific guidance for augmenting social support and enhancing the health of this vulnerable population.

This research intends to provide three key contributions: Initially, it broadens the research scope on health vulnerability by focusing on migrant workers, so enriching the theoretical comprehension of the correlation between temperature fluctuations and health vulnerability within this demographic. Secondly, it ameliorates the assessment of health vulnerability indicators. The study employs the socio-ecological model to assess the influence of temperature change in China in 2020 on the health vulnerability of migrant workers. Finally, the study examines the underlying mechanism of this impact by incorporating psychological burden as a mediating component. This research seeks to furnish policymakers with evidence to more effectively tackle the public health concerns presented by climate change, especially in protecting the health of at-risk groups.

The subsequent sections of this work are structured as follows: Section 2 delineates the literature review. Section 3 delineates the data sources and elucidates the research methodology. Section 4 delineates the empirical analysis. Section 5 delineates the conclusion and discussion. Section 6 delineates the practical implications and managerial insights. Section 7 presents prospective avenues for investigation.

## 2 Literature review

### 2.1 Advancement in health vulnerability indicators research

Health vulnerability denotes the sensitivity, exposure, and adaptation capability of individuals or groups to climate change, reflecting the responsiveness of health outcomes to climate-related dangers and the efficacy of coping strategies (26). The key elements of developing a health vulnerability assessment system is the thorough integration of multidimensional influencing elements and the precision of quantification. Researchers, both nationally and globally, have investigated diverse methodologies for assessing health vulnerability, while the primary dimensions of evaluation frameworks frequently vary. First, a common method for developing indicator systems is based on dimensional composition. Researchers generally consider aspects such as physiological health, environmental factors, and socioeconomic circumstances (27–30). Although these studies include essential aspects such as physiological health, environmental conditions, and socioeconomic status, the complexity and subjectivity of the conclusions may stem from the weighting and interplay of various dimensions. Secondly, regarding measurement tools and methodologies, several academics have developed vulnerability assessment frameworks by considering health determinants and health outcomes as two essential components (31, 32). These frameworks utilize diverse methodologies, including analysis of variance (ANOVA) (31), empirical orthogonal function (EOF) analysis (32), equal weighting methods (33), principal component analysis (PCA) (33, 34), and dimensionality reduction statistical methods (35), among others. Certain scholars have performed evaluations about the origins of health impact exposure, highlighting the detrimental effects of these sources on individual health. These studies rigorously analyze potential health risks linked to exposure sources by examining criteria such as disaster intensity, geographical extent, frequency, and duration. The objective is to investigate the correlation between exposure sources and health outcomes, encompassing morbidity (32, 36), death (31, 36), and individual health status (33). Prevalent methodologies encompass expert assessment (26), case study approaches (36, 37), and more ways. These assessment approaches are relevant across different spatial scales, covering a broad range of subjects, including regions (33, 38), communities (39), and cities (40). The evaluations encompass multiple forms of health impact exposure, including air pollution (41), flooding (33, 34), elevated temperatures (34, 36), and increasing sea levels (37).

Prior research has established a basis for the development of health vulnerability assessment systems; nevertheless, there remains room for further exploration. Primarily, regarding the design of health indicators, the majority of experts have concentrated mostly on physiological wellness (26, 31–37). This research, however, comprehensively examines physiological health, psychological health, and health habits. Both physical and mental health substantially affect vulnerability, whereas health habits determine long-term health consequences. At the macro level, researchers generally take into account geographical or habitat conditions regarding environmental elements. Geographic location influences healthcare accessibility, and environmental quality directly impacts health. Secondly, regarding research perspective, this study concentrates on the migrant worker demographic. Prior research has examined vulnerability from multiple viewpoints, such as the older adult (42–45) and children (46–49), or have examined migrant workers’ health vulnerability. Chinese migrant workers constitute a unique demographic, arising from particular socioeconomic and policy contexts with significant contributions to social development. Nonetheless, scholarly investigation on this cohort remains very nascent. Thirdly, regarding data authority and generalizability, a substantial number of previous studies have depended on questionnaires or interviews administered by research teams focusing on particular regions or populations (26, 36, 37). Although these research provide useful insights, their findings frequently lack generalizability. This study, different from this, utilizes authoritative public databases from nationwide surveys done by reputable institutions, hence augmenting the universality and trustworthiness of the research findings.

### 2.2 Research on the impact of temperature changes and the health of migrant workers

The concept of the “migrant worker” emerged in the early stages of China’s economic reforms as a response to surplus rural labor, reflecting the migration patterns within China’s urban–rural dual structure (50). Current studies on the correlation between temperature variations and the well-being of migrant workers predominantly emphasize the effects of elevated temperatures (51). Many migrant workers, mainly in the construction sector, are subjected to elevated temperatures for extended durations, resulting in heat stress, profuse sweating, weariness, headaches, and diminished work efficiency and social interaction (52). Exposure to elevated temperatures heightens the risk of heat-related ailments, such as heat exhaustion, dehydration, and potentially heatstroke (53). Prolonged exposure to high temperatures can also heighten the risk of acquiring respiratory and cardiovascular problems (54, 55). Moreover, increasing temperatures have been demonstrated to expedite the transmission of some diseases, broadening their geographical range (56). Certain research have investigated the effects of cold temperatures on the health of migratory laborers. Migrant workers in the fishing business encounter increased risks of injury and disease in colder environments (57). During the COVID-19 pandemic, reduced temperatures heightened the vulnerability of migrant workers to the virus within healthcare institutions, as they regularly operated on the frontlines, often interacting with patients and their close contacts (58). The majority of migratory laborers either lack the awareness to acquire or opt not to obtain formal work injury and unemployment insurance to conserve funds, thus, when in instances of health issues resulting from temperature variations, compensation is severely limited (59). This leads to an inability to finance medical care, exacerbating their physical health situation. Moreover, substantial temperature variations intensify the psychological strain on migrant laborers, in addition to their direct effects on physical health. Temperature fluctuations, coupled with occupational stress, frequently result in anxiety and tension, hence heightening the likelihood of mental health disorders (60). Although current research has explored the impact of temperature changes on the health of migrant workers, it predominantly concentrates on certain diseases or psychological stressors. Research focused on the health vulnerabilities of migrant workers concerning temperature variations is limited. Therefore, this paper takes temperature changes as the focal point, specifically examining their impact on the health vulnerability of migrant workers, filling this gap in the literature. This study additionally examines the mechanisms by which temperature change influence the health vulnerability of migrant workers by incorporating psychological burden as a mediating variable, thereby developing an impact mechanism model grounded in relevant theoretical frameworks.

### 2.3 Temperature changes, psychological burden, and health status

Multiple research have underscored the substantial effects of temperature change on both physical and mental health, affected by many factors (4, 61). The mechanisms governing body temperature indicate that mental health may be affected by environmental temperatures, as specific neurotransmitters, including biogenic amines, are involved in mood regulation and thermoregulation (62). Increasing temperatures facilitate the dissemination of vector-borne diseases (63) and aquatic illnesses (64), while simultaneously promoting the proliferation of fungi and molds, hence elevating the incidence of respiratory and asthma-related conditions. Extreme temperatures, whether elevated or reduced, have adverse impacts on both the body and the mind. Besides their physical impact, severe temperatures provide significant psychological difficulties. Increased temperatures, specifically, have been demonstrated to heighten psychological distress, whilst decreased temperatures frequently mitigate certain adverse mental conditions (65). Temperature fluctuations may intensify risk factors for established mental health issues (66), with extended exposure to elevated temperatures linked to increased psychological stress and mental tiredness (67). The impacts are particularly significant among migratory workers, who frequently endure unstable working conditions with restricted access to suitable housing, adequate rest intervals, or opportune psychiatric assistance (53). The interplay of environmental stresses and socioeconomic pressure amplifies psychological vulnerability, worsening mental health conditions (68). Vulnerability is often a matter of perception, as different populations experience and respond to temperature changes differently, leading to varying levels of psychological burden (69). The stress induced by temperature changes compromises the immune system, rendering migrant workers more vulnerable to sickness (70). Furthermore, the psychological burden of coping with extreme temperatures frequently leads to maladaptive coping mechanisms, like heightened alcohol intake or unhealthy eating practices, which exacerbate general health deterioration (71). This research empirically investigates the effects of temperature change on the health vulnerability of migratory workers and explores the underlying mechanisms. The objective is to elucidate the mechanisms by which temperature fluctuations affect health vulnerability, so establishing a scientific foundation for the development of pertinent policies.

### 2.4 Intergenerational health disparities among migrant laborers

The health disparities between older and newer generations of migrant workers have been well documented (72). Temperature has also emerged as an important factor influencing these differences, as research demonstrates that fluctuations in temperature exposure during pregnancy can variably affect the health of subsequent generations, with elevated temperatures exerting a more adverse impact on birth outcomes (73–75). Firstly, significant intergenerational differences exist in the physical health of migrant workers. Research indicates that the older cohort of migrant workers, who migrated to urban areas in the 1980s and 1990s, encountered more unfavorable working circumstances, possessed little health knowledge, and had insufficient access to appropriate healthcare facilities. In comparison to the general population, the absence of prompt healthcare or the neglect to disclose health concerns frequently intensified their health vulnerability (76). The newer generation of migrant workers, who have relocated to urban areas, generally possess elevated educational qualifications and enhanced access to health-related information, so contributing to their overall health improvement (77). Besides physical health, notable intergenerational disparities are also seen in the psychological well-being of migratory workers. The newer generation of migrant workers faces unique challenges due to the effects of China’s economic reforms and the one-child policy (78). Factors include diminished social support, reduced marriage rates, a disparity between expectations and reality, and challenges in urban integration have rendered the psychological well-being of the younger generation more susceptible than that of their older counterparts (79–81).

### 2.5 Contributions to research

In summary, whereas current research has made significant advancements and laid a solid foundation for this study, several research gaps remain to be addressed.

Firstly, the correlation between temperature changes and the health vulnerability of migrant workers remains little investigated. This research improves the assessment of health vulnerability by utilizing micro-level data and developing a health vulnerability index grounded in the social-ecological model, building upon prior studies. Health vulnerability is intricately linked to an individual’s social background and lifestyle choices. Our concept encompasses various health dimensions, including physical health, mental health, habits, social support, and environmental exposure, situating individual health within a wider social and ecological framework. This methodology uncovers the intricate relationships between health and social-ecological factors (82, 83), facilitating a more refined evaluation of the effects of temperature variations on health.

Secondly, this work presents a novel research perspective. Migrant workers merit attention for their substantial contributions to socio-economic development; nonetheless, their socioeconomic position is relatively low. While certain researchers have examined the health vulnerabilities of migrant workers, these studies have primarily focused on international migrant workers. However, within the context of different countries, Chinese migrant workers possess distinct traits and qualities compared to global migrant labor populations. Research on migrant laborers from foreign nations is not universally relevant to China’s distinct situation. This research addresses such deficiency.

Lastly, this research examines both the immediate effects of temperature change on health vulnerability and the underlying mechanisms involved. By investigating how social, economic, and environmental conditions interact with temperature changes, the study provides fresh insights into the pathways through which climate impacts migrant workers’ health vulnerability. Given the significant intergenerational differences between older and newer generations of migrant workers, the study further examines how temperature change differentially affect the health vulnerability of these two groups.

## 3 Research design

### 3.1 Sample selection and data sources

The individual sample data for this study originates from the 2020 China Family Panel Studies (CFPS) conducted by the China Social Science Survey Center at Peking University. Meteorological data is obtained from the National Meteorological Science Data Center of China, which provides daily data on fundamental meteorological elements from more than 2,400 stations nationwide, encompassing temperature, air pressure, relative humidity, precipitation, wind direction and velocity, and sunlight. This study utilizes county-level administrative codes from the CFPS data to identify respondents’ actual residences and use GIS technology to physically correlate meteorological station data with respondents’ locations using latitude and longitude coordinates. In covered by multiple meteorological stations, the average data from these stations is utilized to more precisely represent the regional climate features. Utilizing the methodologies of Currie and Neidell (84), Deschênes and Greenstone (85), and Schlenker and Walker (86), we additionally employed the Inverse Distance Weighting (IDW) technique to interpolate daily meteorological data, thereby improving the continuity and comprehensiveness of the dataset, specifically mitigating potential biases stemming from the irregular distribution of meteorological stations. Furthermore, we consolidated the daily meteorological data into seasonal data and matched it to the city-year CFPS data to maintain consistency across the temporal dimension. We standardized the geographic coding of the CFPS and meteorological datasets to ensure data consistency, thereafter conducting data cleaning and verification. Missing and outlier values were omitted, and the matching logic was verified using random sampling. Ultimately, by integrating macroeconomic data, we acquired 3,930 valid observations at the individual-city level, so ensuring the correctness and scientific rigor of the data.

### 3.2 Variable definitions

#### 3.2.1 Dependent variables

Health Vulnerability (HV). In this study, the health vulnerability index is constructed based on the Social-Ecological Model (SEM) framework using micro-level data (87), and is calculated through the entropy weighting method. The entropy method determines the weights of each indicator by comparing the amount of information provided by the indicators. Due to the small values obtained from the entropy calculation, directly using the raw values could compromise the interpretability and readability of the results. This study employed a moderate scale adjustment by multiplying the indicators by 10,000 to enhance the intuitive presentation of the data, and facilitate the intuitive presentation of the data and the expression of the practical significance of the research findings, while rendering the value range more conducive to analysis (88).

The Social-Ecological Model (SEM) is a multi-tiered theoretical framework for examining individual behaviors, highlighting that such acts are shaped by numerous causes. The Social-Ecological Model (SEM) started from Urie Bronfenbrenner’s ecological systems theory and subsequently developed into a theoretical framework that elucidates how individual health or behavior is affected by various layers of components, including personal, societal, and environmental impacts. It also examines the interplay among these stratified elements. The ecological environment is not autonomous; it results from the interplay between human activities and the natural ecosystem (89). Health vulnerability results not only from environmental pressures but is also intricately linked to human social conduct; for example, famines are frequently instigated by war or other circumstances. Social behaviors exacerbate conflicts about resource allocation among migrant workers and between migrant workers and urban residents, whereas social factors encompass specific human behaviors that affect health vulnerability (18). In the presence of temperature fluctuations, all individuals are vulnerable, but the social repercussions are uneven. Social considerations can reveal which groups are more vulnerable to harm in unfavorable conditions (90). Vulnerability is a question of perception, influenced by socio-economic status, demographic characteristics, and other factors that affect migrant workers’ perception of temperature variations (91). Each environmental issue possesses an own historical background and is situated inside a particular social milieu (92). Consequently, the analysis of health vulnerability must account for the interaction of various dimensions, encompassing individual, societal, and environmental factors. The social-ecological model offers a systematic theoretical foundation for this objective. The Social-Ecological Model (SEM) effectively illustrates the health vulnerability of migratory workers in relation to their sensitivity to exposure losses caused by environmental and social changes. Based on this, this study utilizes SEM to elucidate the impact of individual, behavioral, social, and environmental factors on the health vulnerability of migrant workers, as well as the interactions among these elements.

Individual-level indications represent inherent traits and a comparatively stable condition developed over time, encompassing both physical and mental health. Suboptimal physical fitness may result in heightened sensitivity to temperature fluctuations (93). Self-assessed health indicates a person’s general health condition (94), but the incidence of chronic illnesses effectively quantifies the impact of enduring health challenges (95). Symptoms of physical discomfort directly indicate both short-term and chronic health conditions (96). Physical discomfort and disease exemplify the health susceptibility of migrant workers (97). Depressive symptoms are intricately linked to psychological vulnerability (98), while life satisfaction signifies both mental well-being and quality of life (99). The behavioral level delineates the decisions individuals undertake in their daily lives, illustrating how they actively impact their health. The intake of tobacco and alcohol is frequently correlated with illness prevalence and mortality rates (100, 101). The frequency of exercise and dietary practices strongly influence health outcomes (102, 103). Collectively, these elements influence a person’s susceptibility to external stressors. Social-level indicators relate to the social context and socioeconomic circumstances of individuals. Social disparities are associated with an individual’s capacity to alleviate vulnerability risks (104). Vulnerability is also a manifestation of efficacy of social resource allocation, reflecting the various levels of efficiency of social resource distribution among migrant workers (17). Reduced income and educational attainment are frequently correlated with elevated health risks (105), and social support can mitigate the adverse impacts of socioeconomic stress on health (106). Limited social support results in discrimination and prejudice, thereby exacerbating the vulnerability (107). Environmental-level indicators denote the work environment and geographic location. Extended exposure to detrimental air quality elevates the likelihood of respiratory illnesses and exacerbates overall health risks and socioeconomic challenges stemming from the cumulative impacts of urbanization, industry, and vehicular emissions (108). Areas with diminished healthcare accessibility have reduced capacity to cope with health hazards (109). The availability of healthcare services and their utilization directly influence health outcomes, while health insurance coverage is essential for ensuring individuals obtain necessary medical care (110). The particular indicators selected are detailed in Table 1.

**Table 1:** Health vulnerability indicator system.

Primary dimension	Secondary dimension	Indicator	Data source	Indicator attribute
Individual Level	Physical Health	Self-Rated Health Status	CFPS, Item: Self-Rated Health	−
		Chronic Disease Rate	CFPS, Item: Chronic Illness	+
		Physical Discomfort Symptoms	CFPS, Item: Recent Physical Discomfort	+
	Mental Health	Depression Symptoms	CFPS, Item: CES-D Depression Scale	+
		Life Satisfaction	CFPS, Item: Life Satisfaction Rating	−
Behavioral Level	Lifestyle	Smoking and Drinking Habits	CFPS, Item: Smoking and Drinking Frequency	+
		Exercise Frequency	CFPS, Item: Exercise Frequency	−
		Dietary Habits	CFPS, Item: Dietary Habits	−
Social Level	Socioeconomic Status	Income Level	CFPS, Item: Household Income	−
		Education Level	CFPS, Item: Highest Education Attained	−
		Social Support	CFPS, Item: Social Support	−
Environmental Level	Access to Medical Services	Health Insurance Coverage	CFPS, Item: Type and Coverage of Health Insurance	−
		Availability of Medical Services	National Statistical Yearbook	−
		Frequency of Hospital Visits	National Statistical Yearbook	−
	Environmental Exposure	Work Environment Quality	CFPS, Item: Quality of Work Environment	−

In the baseline regression, the entropy weight technique assesses health vulnerability, whereas the robustness tests utilize principal component analysis (PCA) to quantify health vulnerability. An increased health vulnerability score signifies a heightened level of health susceptibility.

#### 3.2.2 Independent variables

Temperature Change (Temp). Utilizing the methodology of Hsiang et al. (111), we developed seasonal average temperature indicators: spring (Temp_spring), summer (Temp_summer), fall (Temp_fall), and winter (Temp_winter). Seasonal patterns of temperatures yield a more precise assessment of the influence of temperature fluctuations on health vulnerability than yearly averages, owing to seasonal temperature patterns (4). The temperature increase studied in this research refers to the elevation in seasonal temperature values, rather than solely extreme heat. An examination of the current research indicates that the consequences of temperature increase extend beyond extreme heat; moderate elevated temperatures can also influence health (112). Warm winter temperatures have been shown to correspond with specific health burdens (113). Although extreme heat and cold have been extensively researched, the possible negative health impacts of untimely warm weather merit additional investigation. Current study suggests that untimely warm weather may adversely impact health (114).

#### 3.2.3 Control variables

This study incorporates the control variables to guarantee that the model remains unaffected by extraneous causes. The control variables are classified into individual, meteorological, and macroeconomic indicators. The individual-level control variables comprise gender (Gen), age (Age), years of education (Edu), marital status (Mar), family size (FS), and health status (Health). Previous studies have shown that older individuals (115), those with lower levels of education (115), individuals in poorer health (115), men (116), and those from larger households (117) exhibit greater health vulnerability. Married women, due to the additional time and energy required to care for their families, are more vulnerable to temperature fluctuations (118). To consider meteorological influences, weather variables like sunshine duration (Sun), precipitation (Rain), average wind speed (Wind), dew point temperature (Dew), and atmospheric pressure (Pressure) are incorporated (119, 120). At the macroeconomic level, we incorporate regional GDP (GDP), industrial structure (IS), and the ratio of health and social welfare workers per 10,000 population (HSWW per 10 k) to account for overarching socioeconomic conditions.

#### 3.2.4 Mediating variable

Psychological burden (PB) quantifies the mental strain and emotional reactions of individuals confronted with pressures. The data is derived from the CFPS. This scale assesses the severity of depressed symptoms in individuals and is utilized to investigate the mediating mechanism between temperature fluctuations and health vulnerability.

### 3.3 Model

This study utilizes cross-sectional data and employs a multivariable regression model to examine the effects of temperature change on the health vulnerability of migratory workers. This model accounts for both individual and city-level attributes, with standard errors clustered at the individual level. The multivariable regression model is formulated as follows:

(1) $$HV_{u,i}=\alpha_{0}+\beta_{1}Temp_{u,i}+\delta_{v}X_{u,i}+e_{u,i}$$

In Equation 1, the subscript u denotes the city, while i signifies the individual sample. HV represents the dependent variable. $X_{u,i}$ signifies the control variables, encompassing gender, age, years of education, marriage, family size, health status, sunshine duration, precipitation, average wind speed, dew point temperature, atmospheric pressure, regional GDP, industrial structure, and the number of health and social welfare workers per 10,000 population. $e_{u,i}$ denotes the random disturbance term, $\alpha_{0}$ signifies the constant term, $\beta_{1}$ represents the coefficient for the explanatory variable, and $\delta_{v}$ indicates the coefficients of the control variables.

## 4 Empirical analysis

### 4.1 Descriptive statistics

Table 2 displays the descriptive statistics of the variables. The findings indicate that the mean value of HV is 0.3726, accompanied by a standard deviation of 0.2423, signifying considerable variability in individual health vulnerability within the sample. The mean temperatures for spring, summer, autumn, and winter are 14.77°C, 24.26°C, 14.07°C, and 2.12°C, respectively. The winter temperature exhibits the highest standard deviation, indicating the greatest regional variability in winter temperatures. Gen and Mar are binary variables, with means of 0.359 and 0.866, respectively, signifying that 35.9% of the sample is male and 86.6% is married. The mean age is 48.89 years, while the average years of schooling is 8.43 years. The mean FS is 3.65, accompanied by a substantial standard deviation, indicating variability in family sizes within the sample. The average Health is 0.706, suggesting that the majority of persons in the sample self-identify as healthy. Environmental data, including sunshine duration, precipitation, wind speed, dew point temperature, and atmospheric pressure, have been normalized to have averages around 0 and standard deviations near 1. In terms of economic variables, the average regional GDP is 16.69, the mean IS value is 0.60, and HSWW per 10 k has a mean value of 3.55, reflecting the variation in economic and social security levels across different cities.

**Table 2:** Descriptive statistics of variables.

Variables	N	Mean	Std	Min	Max
HV	3,930	0.3726	0.2423	0.0062	1.5382
Temp_spring	3,930	14.7716	4.3651	6.3128	24.7043
Temp _summer	3,930	24.2566	3.3703	16.4003	29.6642
Temp_fall	3,930	14.0688	5.1047	5.6763	25.6294
Temp _winter	3,930	2.1160	7.3982	−16.9848	17.4989
Gen	3,930	0.3590	0.4798	0.0000	1.0000
Age	3,930	48.8850	14.2204	18.0000	88.0000
Edu	3,930	8.4262	5.6594	0.0000	19.0000
Mar	3,930	0.8656	0.3411	0.0000	1.0000
FS	3,930	3.6545	3.7367	−8.0000	15.0000
Health	3,930	0.7059	0.4557	0.0000	1.0000
Sun	3,930	0.0177	0.9997	−2.1633	2.2261
Rain	3,930	−0.0170	0.9953	−1.4819	2.4025
Wind	3,930	0.0078	1.0003	−2.3403	3.3872
Dew	3,930	−0.0165	0.9965	−1.9903	2.2005
Pressure	3,930	−0.0013	1.0032	−2.5042	1.0690
GDP	3,930	16.6945	0.9502	15.2994	19.7740
IS	3,930	0.5996	0.0746	0.3086	0.7315
HSWW per 10 k	3,930	3.5534	3.8311	0.6610	30.9970

### 4.2 Baseline regression and mechanism testing

To evaluate the model’s robustness, we conducted the Pesaran cross-sectional dependence test (Pesaran CD Test) on both the baseline regression model and the mechanism verification model to determine the presence of cross-sectional dependence among the sample units. The *p*-values for the three models were 0.15, 0.21, and 0.22 (Table 3). According to the null hypothesis of the Pesaran test (H0: no cross-sectional dependency) and the *p*-values obtained, we fail to reject the null hypothesis, suggesting the absence of significant cross-sectional dependence in the models. This suggests that there is no significant cross-sectional association in the health vulnerability of migratory workers within the modeling framework of the present study. The random disturbances between different individuals are relatively independent. Consequently, the outcomes from our regression model can utilize two-way fixed effects regression estimates, which possess high robustness and scientific validity, without necessitating further model adjustments to resolve cross-sectional dependence concerns.

**Table 3:** Baseline regression and mechanism testing results.

	(1)	(2)	(3)
Variables	HV	PB	HV
PB			0.0514***
			(12.16)
Temp_spring	0.0958***	0.2822***	0.0279***
	(11.66)	(9.09)	(4.38)
Temp_summer	0.0556***	−0.0342**	0.0075**
	(25.63)	(−2.11)	(2.48)
Temp_fall	−0.1960***	−0.2196***	−0.0586***
	(−36.62)	(−5.35)	(−7.38)
Temp_winter	0.0169***	−0.1856***	0.0066*
	(2.86)	(−12.76)	(1.95)
Gen	0.1020***	−0.1367***	0.1212***
	(10.70)	(−5.41)	(12.16)
Age	0.0030***	−0.0018	0.0031***
	(8.68)	(−1.47)	(9.45)
Edu	0.0002	−0.0007	−0.0003
	(0.27)	(−0.26)	(−0.43)
Mar	−0.0119	0.0292	−0.0038
	(−1.18)	(0.72)	(−0.35)
FS	0.0284***	−0.0083	0.0019
	(18.57)	(−1.10)	(1.23)
Health	−0.1273***	−0.2983***	−0.1252***
	(−12.75)	(−7.49)	(−12.45)
Sun	0.2701***	0.7794***	0.0469***
	(24.62)	(12.53)	(3.76)
Rain	−0.0749***	1.2576***	0.0898***
	(−3.59)	(11.94)	(4.17)
Wind	−0.0333***	−0.1213***	−0.0017
	(−25.08)	(−16.58)	(−1.03)
Dew	0.3610***	0.0202	0.0028
	(10.85)	(0.13)	(0.09)
Pressure	0.0075	0.3163***	0.0686***
	(0.88)	(8.16)	(8.50)
GDP	−0.0268***	−0.2178***	−0.0052*
	(−10.56)	(−23.80)	(−1.99)
IS	1.4892***	2.5864***	0.3829***
	(14.74)	(5.65)	(4.20)
HSWW per 10 k	−0.0321***	−0.0724***	−0.0147***
	(−8.65)	(−5.33)	(−4.94)
Constant	−0.1738	5.0016***	0.4058***
	(−1.02)	(7.64)	(2.88)
id	Control	Control	Control
city	Control	Control	Control
Observations	3,930	3,930	3,930
R-squared	0.267	0.091	0.249
Pesaran CD Test *p*-value	0.15	0.21	0.22

*** Denotes significance at the 1% level, ** denotes significance at the 5% level, and * denotes significance at the 10% level. The figures within parenthesis represent *t*-values.

The baseline regression results (Table 3, Column 1) indicate that increasing spring and summer temperatures significantly enhance health vulnerability, with coefficients of 0.0958 and 0.0556, respectively, both significant at the 1% level. The effect of spring temperature increases is more pronounced. Winter temperature increases exert a notable positive influence on health vulnerability, but to a lesser extent, with a coefficient of 0.0169, significant at the 1% level. Conversely, increasing fall temperatures have a substantial negative correlation with health vulnerability, with a coefficient of −0.1960, which is significant at the 1% level, suggesting that higher fall temperatures markedly diminish health vulnerability. We also account for many additional characteristics that may influence the health susceptibility of migratory workers, including gender, age, years of education, marital status, family size, health status, and sunshine duration. Gender and age are significant at the 1% level, indicating disparities in health vulnerability among migrant workers of varying genders and ages.

The mechanism testing revealed that psychological strain partially mediates the association between temperature fluctuations and the health vulnerability of migrant workers. In model (3) (Table 3, Column 3), the coefficient for the influence of psychological burden on health vulnerability is 0.0514, significant at the 1% level, signifying that an increase in psychological burden correlates with an increase in health vulnerability. In model (2) (Table 3, Column 2), the coefficient for the impact of increasing spring temperatures on psychological burden is 0.2822, significant at the 1% level, indicating that for each unit increase in spring temperature, psychological load rises by approximately 0.282 units. The coefficients for summer, fall, and winter temperatures on psychological burden are −0.0342, −0.2196, and − 0.1856, respectively, and are statistically significant at the 5, 1, and 1% levels. The results indicate that temperature increases over these three seasons alleviate psychological strain, with the greatest significant relief observed with rising temperatures in the fall. Furthermore, sunshine length exerts a substantial positive influence on health vulnerability, evidenced by a coefficient of 0.2701, indicating that extended sunshine durations correlate with increased health vulnerability. This may be associated with heightened stress or other health complications resulting from extended exposure to sunshine.

### 4.3 Heterogeneity test

This study performed a heterogeneity analysis between the new and old generations of migrant workers, with the new generation being individuals born in 1980 or later, and the old generation consisting of those born prior to 1980 (121). The “post-1980 migrant workers,” born in the 1980s and 1990s, were classified according to the pivotal historical setting of China’s reform and opening-up, which commenced approximately at that period, is also known as the contemporary industrial workers and rural builders. The living experiences of this generation significantly diverge from those of their parents, influenced by swift economic advancement, more global engagement, the implementation of the one-child policy, and the proliferation of the internet and globalization. The “new generation of migrant workers” denotes a subset of the “post-80s” cohort, characterized by their distinct life experiences—lacking substantial agricultural background, reluctant to reside permanently in rural locales, yet finding it difficult to change their rural identity and encountering challenges in assimilating into urban society while enduring uncomfortable living conditions (122). Their experience of migrating to cities contrasts differs from that of the previous migrant workers who relocated to cities during the initial stage of China’s reform. The current generation has unique demographic compositions, social attitudes, identity dimensions, and work-life aspirations (123). Table 4 displays the outcomes of the heterogeneity test. The findings indicate that the coefficients representing the effect of increasing spring temperatures on the health vulnerability of both new and old generation migrant workers are 0.0444 and 0.1249, respectively, with both being statistically significant at the 1% level. This suggests that increasing spring temperatures markedly elevate health susceptibility for both cohorts, with a diminished effect on the younger generation. The coefficients indicating the impact of increasing summer temperatures on health vulnerability are 0.0320 for the younger generation and 0.0175 for the older generation, both statistically significant at the 1% level. This indicates that increasing summer temperatures intensify health risk, particularly affecting the new generation migrant workers more significantly. In the autumn, the coefficients indicating the effect of increasing temperatures on health vulnerability are −0.1030 for the younger generation and − 0.1451 for the older generation, both statistically significant at the 1% level. This suggests that increasing fall temperatures reduce health vulnerability, particularly benefiting older migratory workers. In the case of winter temperatures, the coefficients are negative for both groups, but only statistically significant for the new generation, with a coefficient of −0.0204 at the 10% level. This suggests that rising winter temperatures have a mild alleviating effect on the health vulnerability of the new generation, though the effect is relatively weak.

**Table 4:** Heterogeneity test results.

	(1)	(2)
	First generation migrant workers	New generation migrant workers
Variables	HV	HV
Temp_spring	0.1249***	0.0444***
	(10.17)	(4.81)
Temp_summer	0.0175***	0.0320***
	(3.29)	(3.79)
Temp_fall	−0.1451***	−0.1030***
	(−20.37)	(−6.82)
Temp_winter	−0.0221	−0.0204*
	(−1.51)	(−1.90)
Gen	0.1077***	0.0896***
	(10.30)	(5.69)
Age	0.0020***	0.0022**
	(3.38)	(1.99)
Edu	0.0003	−0.0031
	(0.38)	(−1.55)
Mar	−0.0049	−0.0062
	(−0.28)	(−0.34)
FS	0.0311***	0.0080***
	(20.74)	(2.65)
Health	−0.1316***	−0.1077***
	(−10.49)	(−4.23)
Sun	0.2410***	0.2248***
	(19.71)	(10.51)
Rain	0.0841***	0.0228
	(25.85)	(0.91)
Wind	−0.0237***	−0.0249***
	(−19.83)	(−5.89)
Dew	0.1850***	0.3597***
	(6.33)	(7.51)
Pressure	0.0562***	0.0580***
	(18.33)	(5.77)
GDP	−0.0259***	−0.0419***
	(−15.92)	(−9.91)
IS	1.3702***	1.2295***
	(10.85)	(9.39)
HSWW per 10 k	−0.0454***	−0.0345***
	(−6.73)	(−5.83)
Constant	−0.1276	0.6170***
	(−0.66)	(3.56)
id	Control	Control
city	Control	Control
Observations	2,761	1,169
R-squared	0.311	0.200

*** Denotes significance at the 1% level, ** denotes significance at the 5% level, and * denotes significance at the 10% level. The figures within parenthesis represent *t*-values.

### 4.4 Robustness tests

Table 5 displays the outcomes of the robustness tests. To validate the empirical findings, two kind of robustness checks were performed: (1) Replacing the Dependent Variable: In the robustness assessments, health vulnerability was determined utilizing the principal component analysis (PCA) technique. The findings demonstrate that spring, summer, and winter temperatures have a notably positive impact, whereas fall temperatures dramatically diminish health vulnerability. These data indicate that the influence of seasonal temperature variations on health vulnerability remains consistent even after altering the dependent variable. (2) Modifying the Model: The second robustness test utilized a multilevel model to consider both individual-level and city-level influences. In contrast to the baseline regression model, the multilevel approach is more suitable for managing hierarchical data structures, such as the layered relationships between cities and individuals. It encapsulates the interplay between individual and urban attributes, yielding more precise estimates and accounting for correlations among individuals at the same level, thus minimizing estimation errors (124). The findings indicate that the impacts of spring, summer, and winter temperatures are no longer substantial, whereas autumn temperature persists in exerting a major negative influence. Control factors, including gender, age, and family size, exhibit comparable levels of significance in both models. Variables such as health status and cumulative precipitation maintain consistent signals and significance across both robustness assessments, hence affirming the validity of the initial findings.

**Table 5:** Robustness test results.

	(1)	(2)
	Replacing the dependent variable	Multi-level model
Variables	HV	HV
Temp_spring	0.0524***	0.0113
	(5.32)	(1.54)
Temp_summer	0.0437***	0.0002
	(9.26)	(0.02)
Temp_fall	−0.1587***	−0.0312**
	(−13.26)	(−2.57)
Temp_winter	0.0426***	0.0124
	(7.95)	(1.61)
Gen	0.0704***	0.1017***
	(5.94)	(13.99)
Age	0.0015***	0.0031***
	(4.63)	(10.35)
Edu	−0.0025***	0.0003
	(−2.92)	(0.50)
Mar	0.0777***	−0.0139
	(5.86)	(−1.33)
FS	−0.0012	0.0281***
	(−0.53)	(27.26)
Health	−0.2690***	−0.1256***
	(−20.31)	(−16.06)
Sun	0.1886***	0.0053
	(10.00)	(0.42)
Rain	−0.0321	0.0296**
	(−1.01)	(2.18)
Wind	−0.0206***	−0.0005
	(−9.16)	(−0.08)
Dew	0.2148***	−0.0419
	(4.36)	(−1.13)
Pressure	−0.0151	0.0409*
	(−1.28)	(1.91)
GDP	−0.0479***	−0.0009
	(−14.09)	(−0.09)
IS	0.7088***	0.0726
	(4.96)	(0.87)
HSWW per 10 k	0.0027	0.0003
	(0.58)	(0.14)
Constant	0.6617***	−1.5603***
	(3.12)	(1.49)
id	Control	Control
city	Control	Control
Observations	3,930	3,930
R-squared	0.281	

*** Denotes significance at the 1% level, ** denotes significance at the 5% level, and * denotes significance at the 10% level. The figures within parenthesis represent *t*-values.

## 5 Conclusions and discussion

Based on the internal mechanisms of how temperature changes affect the health vulnerability of migrant workers, this study introduces psychological burden as a mediating variable and constructs a model to examine the impact of temperature changes on health vulnerability. We developed the health vulnerability index based on 3,930 individual city-level observations from China in 2020, utilizing a multivariable regression model within the social-ecological framework. We assess the influence of temperature change on the health susceptibility of migrant workers and the mediation effect of psychological burden. Ultimately, we reach the subsequent conclusions.

1. Temperature changes exacerbate the health vulnerability of migrant workers, chiefly via augmenting psychological burden. Extended exposure to elevated temperatures, especially among outdoor laborers, activates the hypothalamic–pituitary–adrenal (HPA) axis, resulting in heightened release of stress hormones such cortisol (125). This physiological response impacts the body’s heat stress mechanism and exacerbates psychological strain, heightening emotional issues such as anxiety and sadness (126). As psychological stress escalates, the immune system may become compromised, diminishing the body’s capacity to adapt to temperature fluctuations and increasing health vulnerability.
2. This study develops seasonal average temperature indicators, with varying effects of temperatures across the four seasons on health vulnerability. The empirical analysis presented in this research produces conclusions that diverge from prior studies. We present the research findings as follows:

Spring temperatures exacerbate the health vulnerability of migratory workers, chiefly due to heightened psychological burden. Conventional research indicates that spring and summer often mitigate anxiety symptoms (127), although this study offers an alternative finding: in 2020, spring temperatures exacerbated health vulnerability among migrant workers. The climate in 2020 exhibited extraordinary volatility, characterized by considerable temperature variations, with spring temperatures falling below anticipated levels, diverging from the norm of previous years (128). The onset of the COVID-19 pandemic in early 2020, along with atypical spring temperatures, constituted a dual stressor (129). The pandemic’s travel limitations resulted in numerous migrant workers being stranded in their hometowns or rendered unemployed owing to business closures, exacerbating their fear and instability amid these significant social shifts. The economic instability and company shutdowns during the epidemic imposed additional psychological strain on the migrant labor demographic (130). Theory of group behavior posits that individual emotions and actions can be significantly magnified within a collective context. Migrant workers, as a unique demographic, faced increased anxiety during the pandemic, which was exacerbated by group interactions, resulting in collective panic that intensified individual psychological distress. Migrant workers encountered limited healthcare resources throughout the pandemic, a challenge faced globally. During the pandemic’s peak, the scarcity of medical resources resulted in insufficient health protection for many individuals, hence intensifying their health vulnerabilities (131). Social-ecological theory posits that an individual’s health is affected by many degrees of environmental influences. The combined impact of social pressures from the pandemic, inclement weather, and economic hardships has exacerbated the psychological and physical stresses of migrant workers, hence heightening their health risk.

Although increasing summer and winter temperatures reduced certain psychological stressors, they worsened the overall health vulnerability of migrant workers. This can be explained by the unique seasonal migration patterns of Chinese migrant workers. To explain the reduction in psychological stress during the summer and winter seasons, we draw upon social support theory. The household registration limitations has made ideal compulsory education inaccessible to the children of migrant workers in many cities. Consequently, they frequently choose to have their children undergo mandatory education in their localities instead of financing education at their workplaces, leading to prolonged separation from their children. During summer vacation, migrant workers generally invite their children to visit them in the city of their employment (132, 133). This arrangement offers them emotional support from their families, acting as a buffer against the loneliness and stress encountered while working in urban environments. Family reunions enhance psychological resilience, alleviating the mental strain of professional life. Fortunately, the policy permitting the children of migrant workers to enroll in local schools with their parents has been adopted in provinces like Zhejiang and is progressively being advocated nationwide (134). Similarly, during the Chinese New Year, migrant workers return to their hometowns to reunite with their families (135), further alleviating psychological pressure, especially the stress experienced during the winter in urban environments. Nonetheless, although these reunions provide psychological respite, severe temperatures intensify the physical strain experienced by migratory workers, resulting in heightened health vulnerability. Elevated temperatures lead to health issues, including heat stress and dehydration (136, 137). Research indicates a positive association between increasing summer temperatures and mortality rates (138). Migrant workers frequently abstain from acquiring cooling devices due to financial limitations, rendering them susceptible to elevated summer heat (139). Furthermore, respiratory diseases exhibit greater sensitivity to the adverse effects of elevated temperatures than other ailments (140), and the circumstances are even more detrimental for migratory workers during the COVID-19 pandemic. Intense summer heat presents a significant risk to the well-being of migrant workers, exacerbating their health challenges. Current study has shown validated that even little increases in winter temperatures do not mitigate the health detriments induced by cold conditions (141). Nonetheless, the detrimental health impacts of winter warming—should the frequency of warm winter days rise—may offset the reduction in death and morbidity (114). Moreover, extensive winter heating cold regions in northern areas directly contributes to the decline of air quality, resulting in heightened mortality rates from cardiovascular and respiratory ailments, particularly affecting economically disadvantaged populations (142, 143). This study’s conclusions regarding the effects of increasing winter temperatures align with those of prior studies. During winter, decreased temperatures facilitate the transmission of COVID-19 (144), hence exacerbating the health vulnerability of migrant workers. Our results concerning the influence of increasing winter and summer temperatures on health vulnerability are consistent with prior studies. While family reunions in summer and winter offered social support that alleviated the psychological stress of migratory workers, the physiological stress from increasing temperatures persists, resulting in a combined impact of psychological and physical strain. This contradiction provides brief psychological respite; yet, migrant workers encounter increased physical health issues.

Decreasing autumn temperatures alleviated psychological burden and decreased health vulnerability among migrant workers. Prior studies indicate that autumn droughts intensify food insecurity, heightening vulnerability (145), and that depressed symptoms typically deteriorate during the autumn and winter months (127). Conversely, our study concludes that increasing fall temperatures reduce psychological stress and enhance health outcomes. The temperate conditions of autumn and the serene nature surroundings facilitate stress alleviation. According to psychological restoration theory, natural locations and conducive temperatures foster a serene and comfortable atmosphere (146), hence facilitating psychological recuperation and alleviating the anxiety encountered by migrant workers in metropolitan settings. Many migrant workers struggle with inadequate housing conditions in cities (147). In contrast to the summer heat and winter chill, the temperate autumn climate diminishes dependence on heating or cooling systems, so enhancing living conditions. Furthermore, the low temperatures in fall do not facilitate the transmission of COVID-19 (148). Prevalent health-threatening conditions, such as cardiovascular and respiratory disorders, are less prone to exacerbation during the autumn season (55). During this moderate season, migrant workers encounter reduced physical discomfort, and their enhanced living conditions contribute to a decrease in health vulnerability. Consistent autumn temperatures enhance sleep quality, which in turn decreases anxiety and stress levels. This affirmative feedback alleviates the psychological strain on migrant workers, thereby diminishing their health susceptibility. For migratory workers residing in limited conditions, the moderate fall temperatures provide increased ventilation via open windows, so boosting air circulation, diminishing the risk of disease transmission, and eventually enhancing their overall health.

1. The influence of temperature change on the health susceptibility of migrant workers differs between older and newer generations. Increasing spring temperatures heighten health vulnerabilities for both groups, with the elder generation of migratory workers being more adversely impacted. This is probably attributable to the older generation’s inferior physical condition relative to the younger generation (76), together with their more arduous working and living situations, which diminish their capacity to adjust to climate changes. Conversely, the younger generation is less impacted owing to improved living conditions and enhanced coping resources. Both the new and old generations of migrant workers are considerably affected by increasing summer temperatures, however the impact on the health of the new generation is more pronounced. This may result from variations in working conditions, since companies tend to recruit younger individuals for outdoor or physically strenuous positions, so subjecting them to increased hazards linked to intense heat. Decreasing autumn temperatures adversely impact the health vulnerabilities of both generations, suggesting that moderate fall temperatures may mitigate health risks, particularly favoring the older adult population. As previously noted, this may be linked to their generally poorer living conditions. The issue of inadequate housing is more common among the older generation, meaning they derive greater benefit from the cooler climate. Winter temperatures do not substantially impact the health vulnerability of the older generation, but they do have a mitigating effect on the younger generation. The younger generation may possess enhanced access to social networks and healthcare, enabling them to acquire superior health knowledge and medical resources to address health challenges during the frigid winter, whereas the old generation, potentially devoid of such resources, does not experience a substantial impact.

## 6 Practical significance and managerial implications

This study demonstrates, using empirical analysis, that temperature change intensify the health vulnerability of migrant workers, possessing significant practical implications. First, migrant workers are a crucial impetus for China’s socio-economic advancement; they tolerate uncomfortable working and residential conditions for the advancement of society, with the representation of Chinese nation’s resilience and enduring spirit, which, rendering them more vulnerable to health concerns linked to climate change. This study employs micro-level data and a health vulnerability assessment grounded in the social-ecological model to precisely identify the health risks encountered by migrant workers due to temperature fluctuations, thereby offering data support and decision-making insights for targeted public health interventions. Secondly, the study investigates the process by which temperature change influence the psychological burden of migrant workers, revealing how temperature affects health vulnerability via psychological stress. It affirms the essential significance of mental health in addressing climate change. The study offers practical insights for policymakers by analyzing how temperature change impact the health vulnerabilities of migrant workers. This understanding facilitates the development of health protection strategies that more effectively meet the needs of migratory workers, and increasing societal attention to the health challenges this group faces under climate change conditions.

Based on this research, several policy recommendations are proposed:

1. Individual and behavioral dimensions: Firstly, health monitoring and the prevention of occupational diseases are essential. Governments and employers must consistently offer health assessments, emphasizing early detection and prevention of chronic diseases and occupational ailments. Secondly, migrant workers ought to proactively enhance their health literacy. Health promotion initiatives and complimentary seminars should be conducted in migrant worker congregations, including factory residential areas, urban villages, and small guesthouses, alongside the distribution of comprehensible health brochures to enhance awareness of health management and augment their management skills. Furthermore, “climate change” ought to be incorporated into safety training for migrant workers, instructing them on the fundamentals of climate change and providing them with the information to safeguard themselves against health risks associated with climate change (149). With these routes established, migrant workers ought to improve their awareness of proactively requesting aid and obtaining health information. Additionally, they can proactively purchase flexible employment insurance and business insurance to alleviate health-related concerns. Thirdly, psychological health care customized for the need of migratory workers should be offered. This may involve popularizing the exsiting free “mental health hotlines 12356”, implementing stress management programs, or providing psychological counseling services at workplaces, and actively promote these services, to mitigate the psychological stress and health dangers linked to temperature fluctuations. Lastly, the enhancement of skills and the optimization of employment structures are vital. Migrant workers ought to be incentivized to engage in vocational training to enhance their abilities and facilitate their shift to positions demanding greater technical proficiency and superior working circumstances. This approach helps reduce health vulnerability due to adverse working environments while also increasing income and social adaptability, thereby enhancing resilience to temperature changes (150).
2. Social dimension: First, enhancing the reform of the household registration system and continuing to promote the new urbanization process is essential. The government should not just target elite individuals in its settlement programs but should instead evaluate a comprehensive range of skill traits, allowing migrant workers to access greater social welfare and support. This would alleviate the psychological strain resulting from marginalization in metropolitan environments. Secondly, the social service system needs to be continuously developed. Establishing a robust fiscal and taxing framework, along with an adaptable development model, is crucial, emphasizing the enhancement of urban resilience to temperature fluctuations. This entails the establishment of a risk assessment methodology for climate change adaptation and associated emergency finances. Furthermore, it is essential to facilitate the integration of urban and rural public services, establish dynamic management systems for the health of the transient population, and transition from static to dynamic management approaches. This must rely on thorough and precise data regarding migratory workers and their attributes in the destination regions, facilitating the creation of specialized health management services. Utilizing the internet and 5G technology to enhance health awareness campaigns is essential. Targeted health education initiatives must be executed to dismantle linguistic and cultural barriers between migrant workers and local inhabitants, hence improving the accuracy of care provision. Furthermore, creating a cooperative financial contribution system within provincial authorities and developing transfer payment agreements that correspond with the public health service requirements of the migratory population will alleviate the financial strain on receiving regions (151). Fortunately, the Opinions on Further Strengthening Services and Protection for Migrant Workers, released by the Ministry of Human Resources and Social Security along with nine other departments, delineate various methods to augment support for migrant workers (152, 153). These encompass enhancing skills training and enabling the urban resettlement of those inclined to relocate. It is hoped that these sound measures can be comprehensively executed across the nation as soon as posssible to advantage all migrant workers.
3. Environmental dimension: Firstly, enhancing the working and residential conditions of migrant workers continuously. The government and enterprises should prioritize enhancing occupational and residential conditions. This encompasses the provision of adequate shade apparatus, cooling systems, and heating facilities. Expanding the availability and provision of affordable housing and enhancing initiatives to advocate for low-rent housing is necessary. The government can enhance multi-agent supply and multi-channel security for cheap housing by using market processes, thereby rectifying inefficiencies and inadequate supply in government-provided housing. Secondly, advocating for ecological change and climate adaption strategies is essential. Businesses are advised to expedite their green transformation, decrease carbon emissions, and mitigate environmental stress resulting from temperature fluctuations. Buffer zones and enhanced greenery should be integrated into industrial property to alleviate the detrimental impacts of heat sources in industrial parks on migrant workers (154). The government ought to execute climate adaptation strategies, advocating for energy-efficient architectural designs, including vertical greening, cool roofs, and light-colored roofing, to mitigate health risks linked to extreme weather phenomena. Thirdly, enhancing the accessibility of healthcare and social services, and increasing efforts to promote awareness of new healthcare policies are crucial. The government ought to improve healthcare services and the social security system for migrant workers. This can be accomplished via mobile medical stations, community health centers, and other efforts that offer complimentary health assessments and fundamental medical services. In urban areas with a significant migrant demographic, initiatives should focus on developing a data-driven, technology-enhanced, learning-oriented primary healthcare system for migrants. The electronic medical records system in these environments must be connected with those utilized by secondary and tertiary hospitals, enabling migrant workers to access more convenient medical care for minor conditions (155).
4. Targeted Interventions for Distinct Seasons: Spring is a critical period for epidemics, necessitating government coordination to enhance the availability of medical resources and optimize their allocation efficiency. Furthermore, psychological counseling services for migrant workers should be enhanced throughout the spring to offer essential emotional support. Initiatives to enhance understanding of current social security programs for migrant workers should be strengthened to mitigate their dual burden of psychological and physical pressures. In light of the heightened health hazards associated with elevated summer temperatures, the implementation of more stringent heat protection measures is important, including the provision of sufficient rest intervals and cooling amenities. Furthermore, the training for summer heat emergency response should be improved. Implementing measures such as establishing pocket parks and incorporating vertical greening is essential to harness the cooling benefits of landscaped areas. During severe weather events, the coordination of public facility operations is essential. Moreover, subsidies should be allocated to migrant workers for the acquisition of cooling apparatus, such as air conditioners, to promote their utilization of these devices. In the autumn season, the government and communities are urged to utilize this time to assist migrant workers in restoring their mental health through community initiatives and psychological support programs. During winter, when temperatures decrease, it is advisable to ensure sufficient heating provisions while enhancing health surveillance for migrant workers, particularly for ailments that are more common in this season. Additionally, seasonal-specific meals can be offered to migrant workers, including cooling foods such as mung beans and melons in summer, and warming dishes like Angelica, ginger, and lamb soup in winter.
5. Targeted Strategies Informed by Generational Disparities: For the elder generation of migratory workers, it is essential to fortify health screenings, disseminate health knowledge, and improve the management of chronic diseases. The promotion of telemedicine services should be advocated, alongside measures to engage older adult migrant workers in social activities. Furthermore, structuring sports programs tailored for middle-aged and older migrant workers can enhance sports participation, providing opportunities for physical exercise and boosting overall fitness. Vaccination campaigns should be advocated for the newer generation of migrant workers, accompanied by the dissemination of health information, particularly regarding the avoidance of sexually transmitted infections, communicable diseases, and mental health concerns. Furthermore, the promotion of sleep health education is essential to foster healthy sleep practices, and training in first aid, including CPR and trauma treatment, should be offered. The utilization of health-related mobile applications should be promoted.

## 7 Outlook

1. This analysis examines all migrant laborers in China in 2020 without regional differentiation. Given the substantial climatic variations throughout China’s diverse geographical regions, future study may focus on regional analyses to investigate the impact of temperature change on the health vulnerabilities of migrant workers in distinct geographic contexts. This would elucidate the interaction between larger social and environmental elements and diverse regional situations, either intensifying or alleviating health vulnerability.
2. This study utilizes data from 2020, a year characterized by the COVID-19 pandemic, so imparting real-world relevance to the findings. Future research may be enhanced by integrating multi-year data, facilitating comparisons of migrant workers’ health vulnerability during non-pandemic years. This would facilitate the evaluation of health vulnerability under normal conditions and its correlation with climate factors.
3. The most recent data utilized in this analysis is from 2020, owing to data availability constraints. In the future, as new data emerges, additional research may investigate the lagged impacts of extensive epidemics on health susceptibility. This would yield more comprehensive insights into the effects of pandemics on health outcomes across time and may reveal significant long-term trends.

## Data Availability

The original contributions presented in the study are included in the article/supplementary material, further inquiries can be directed to the corresponding author.
